# Decision Regret Following the Choice of Surgery or Active Surveillance for Small, Low-Risk Papillary Thyroid Cancer: A Prospective Cohort Study

**DOI:** 10.1089/thy.2023.0634

**Published:** 2024-05-24

**Authors:** Anna M. Sawka, Sangeet Ghai, Lorne Rotstein, Jonathan C. Irish, Jesse D. Pasternak, Eric Monteiro, Janet Chung, Afshan Zahedi, Jie Su, Wei Xu, Jennifer M. Jones, Amiram Gafni, Nancy N. Baxter, David P. Goldstein

**Affiliations:** ^1^ Division of Endocrinology, University Health Network and University of Toronto, Toronto, Canada.; ^2^ Joint Department of Medical Imaging, University Health Network-Mt Sinai Hospital-Women's College Hospital, University of Toronto, Toronto, Canada.; ^3^ Department of Surgery, University Health Network and University of Toronto, Toronto, Canada.; ^4^ Department of Otolaryngology-Head and Neck Surgery/Surgical Oncology, Princess Margaret Cancer Centre, University Health Network, Toronto, Canada.; ^5^ Department of Otolaryngology and Head and Neck Surgery, Mount Sinai Hospital and University of Toronto, Toronto, Canada.; ^6^ Department of Otolaryngology and Head and Neck Surgery, Trillium Health Partners and University of Toronto, Toronto, Canada.; ^7^ Division of Endocrinology, Women's College Hospital and University of Toronto, Toronto, Canada.; ^8^ Department of Biostatistics, Princess Margaret Cancer Centre, University Health Network, Toronto, Canada.; ^9^ Department of Biostatistics, Dalla Lana School of Public Health, University of Toronto, Toronto, Canada.; ^10^ Department of Psychosocial Oncology, University Health Network and University of Toronto, Toronto, Canada.; ^11^ Centre for Health Economics and Policy Analysis, Department of Health Research Methods, Evidence, and Impact, McMaster University, Hamilton, Canada.; ^12^ Melbourne School of Population and Global Health, University of Melbourne, Melbourne, Australia.

**Keywords:** papillary thyroid cancer, active surveillance, thyroidectomy, prospective study, decision regret

## Abstract

**Background:**

It is important to understand cancer survivors' perceptions about their treatment decisions and quality of life.

**Methods:**

We performed a prospective observational cohort study of Canadian patients with small (<2 cm) low-risk papillary thyroid cancer (PTC) who were offered the choice of active surveillance (AS) or surgery (Clinicaltrials.gov NCT03271892). Participants completed a questionnaire one year after their treatment decision. The primary intention-to-treat analysis compared the mean decision regret scale total score between patients who chose AS or surgery. A secondary analysis examined one-year decision regret score according to treatment status. Secondary outcomes included quality of life, mood, fear of disease progression, and body image perception. We adjusted for age, sex, and follow-up duration in linear regression analyses.

**Results:**

The overall questionnaire response rate was 95.5% (191/200). The initial treatment choices of respondents were AS 79.1% (151/191) and surgery 20.9% (40/191). The mean age was 53 years (standard deviation [SD] 15 years) and 77% (147/191) were females. In the AS group, 7.3% (11/151) of patients crossed over to definitive treatment (two for disease progression) before the time of questionnaire completion. The mean level of decision regret did not differ significantly between patients who chose AS (mean 22.4, SD 13.9) or surgery (mean 20.9, SD 12.2) in crude (*p* = 0.730) or adjusted (*p* = 0.29) analyses. However, the adjusted level of decision regret was significantly higher in patients who initially chose AS and crossed over to surgery (beta coefficient 10.1 [confidence interval; CI 1.3–18.9], *p* = 0.02), compared with those remaining under AS. In secondary adjusted analyses, respondents who chose surgery reported that symptoms related to their cancer or its treatment interfered with life to a greater extent than those who chose AS (*p* = 0.02), but there were no significant group differences in the levels of depression, anxiety, fear of disease progression, or overall body image perception.

**Conclusions:**

In this study of patients with small, low-risk PTC, the mean level of decision regret pertaining to the initial disease management choice was relatively low after one year and it did not differ significantly for respondents who chose AS or surgery.

## Introduction

Contemporary management of low-risk papillary thyroid cancer (PTC) has been de-escalated and patient treatment preferences should be considered. Active surveillance (AS) of low-risk papillary microcarcinoma was first introduced as an alternative to traditional surgical treatment in Japan in the 1990s.^1,2^ Published research on the long-term clinical outcomes of AS for small, low-risk PTC has grown internationally.^3–14^

In 2016, we introduced AS as an alternative to immediate surgery within a Canadian prospective research study on disease management choice in patients with small, low-risk PTC.^15^ We have previously reported on baseline variables associated with patients' choice of AS or surgery, including fears relating to thyroid cancer progression or surgery.^16^ This study builds on our prior work, in its aim to examine the level of decision regret about a year after our patients made the decision to accept immediate surgery or AS. Decision regret is a distressing emotion, which may be experienced following a life decision, and it may be associated with negative patient perceptions regarding decision satisfaction, quality of life, health outcomes, and health care system experience.^17^ We also examined the level of fear of disease progression, anxiety, depression, quality of life, and perceived body image relative to treatment choice, at one-year follow-up.

## Methods

### Participant eligibility and enrollment

Consenting adult patients (18 years or older), with small (<2 cm maximal diameter), low-risk, untreated PTC who met previously reported eligibility criteria,^15,16,18^ were offered the choice of AS or surgery at the University Health Network, in Toronto, Canada (Clinicaltrials.gov NCT03271892). The disease management options were explained by the investigators verbally and patients were provided a written pamphlet (see Supplementary Data). Patients were informed that surgery was the traditional standard of care for management of PTC in Canada. The patients consented to short-term follow-up regarding their treatment choice and long-term follow-up by medical record review for ultimate treatments and outcomes. Patients consenting to AS agreed to be followed at the study center by a study investigator (A.M.S. or D.P.G.). Consent was also obtained for a one-year patient-reported questionnaire follow-up.

The patients who chose surgery, or those who crossed over to surgery (after initially choosing AS), received the usual postoperative care and follow-up by their own surgeons or endocrinologists. For patients undergoing AS, surgery was recommended for disease progression, but patients without disease progression could cross over to surgery at any time as per their personal choice.^15^

The Research Ethics Board of University Health Network approved the original Toronto study (15-8942)^15^ and a long-term follow-up protocol was approved by the Ontario Cancer Research Ethics Board (ID #1986),^19^ as part of an ongoing, expanded pan-Canadian study (Clinicaltrials.gov NCT04624477).^19^ Informed written consent was obtained from participants, with ethics board-approved modifications in recruitment procedures during the COVID-19 pandemic.^18^

### Study outcomes and data collection

Participants were asked to complete a written questionnaire one year after deciding AS or surgery. This questionnaire included the following components: thyroid cancer treatment status, the Decision Regret Scale,^20^ the Fear of Progression Questionnaire—Short Form questionnaire^21,22^ (focused on thyroid cancer disease progression), the Hospital Anxiety and Depression Scale (HADS),^23^ the MD Anderson Symptom Inventory for thyroid cancer (MDASI-Thy, disease-specific quality of life questionnaire),^24^ and the Body Image Scale (BIS) (a questionnaire on body image perception for cancer patients).^25^ Decision Regret Scale overall score was the primary outcome and this questionnaire is scored on a scale of 0–100, where 100 represents maximal regret.^20^

Although there is no established cutoff for clinically meaningful decision regret, a systematic review suggested that a score of 25 could be used to distinguish low versus higher decision regret.^17^ Fear of disease progression^21,22^ was evaluated in the context of the thyroid cancer diagnosis, at the time of enrollment.^16,26^ The total score on the Fear of Progression—Short Form questionnaire may range from 5 to 60 (where 60 is the worst possible fear).^21, 22^ Permission was obtained from the developers for use of the questionnaires (where applicable) and we scored the questionnaires as per the developers' instructions. We reviewed medical records to confirm clinical data and outcomes.

### Statistics

We summarized descriptive statistics as numbers with percentages or means/medians with standard deviations (SDs)/interquartile ranges. The primary intention-to-treat analysis described the level of decision regret according to the initial disease management choice (AS or surgery), presenting both the crude data and results adjusted for age, sex, and duration of follow-up. For univariate group comparisons, we utilized chi-squared or Fisher's exact tests for categorical data or Wilcoxon rank-sum tests for continuous data. We used linear regression for adjusted analyses, incorporating adjustments for age, sex, and duration of follow-up. The results of linear regression analyses were expressed as beta coefficients with confidence intervals (CIs). Intention-to-treat group comparisons were made according to initial treatment choice (AS or surgery). In secondary *post hoc* analyses, we compared patients who were continuing AS at the time of the questionnaire completion with those who chose surgery or with those who crossed over from AS to surgery, respectively.

We compared baseline and one-year Fear of Progression—Short Form questionnaire scores using a paired *t*-test. The statistical analyses were performed using R 4.1.0 and SAS 9.4 (by the statisticians J.S. and W.X.). Missing data were excluded. The alpha level for statistical significance was set at *p* < 0.05.

## Results

### Description of study participants

**FIG. 1.**
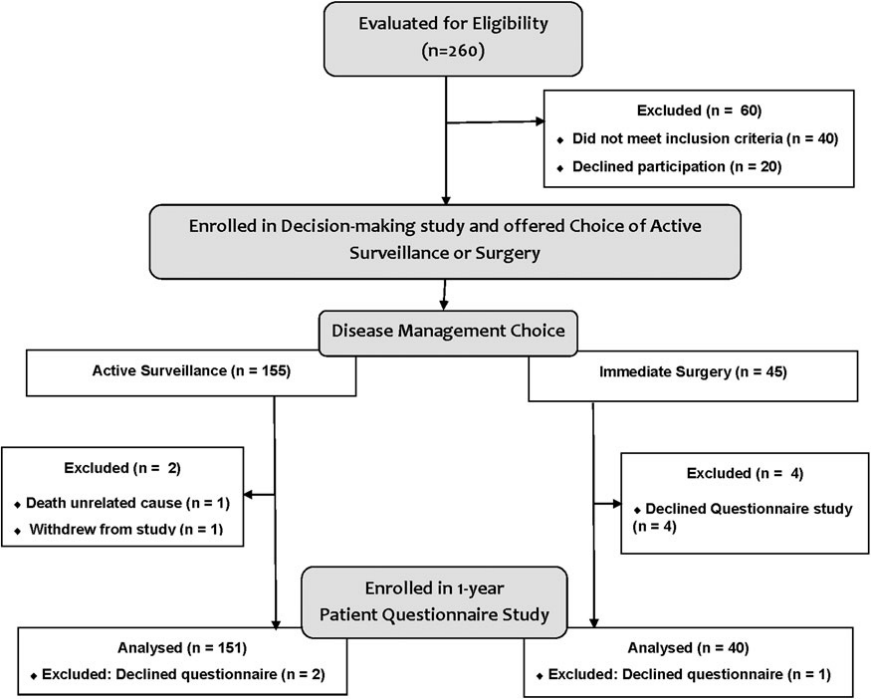
Participant flow diagram.

The participant questionnaire response rate was 95.5% (191/200) of all patients who had been offered the option of AS or surgery in the decision-making study (Fig. 1). Of the nine nonrespondents, one patient in the AS group died of an unrelated cause (pneumonia), one patient in the AS group withdrew from the study before the 1-year follow-up, and four patients from the surgical arm declined participation/consent for the questionnaire follow-up study (Fig. 1). Approximately three-quarters of respondents were women (77%, 147/191). The mean age was 53 years (SD 15 years). The mean baseline primary tumor size was 11 mm (SD 4). The percentage of respondents who initially chose to undergo AS was 79.1% (151/191). A detailed description of the demographic and clinical characteristics of participants is reported in Table 1.

**Table 1.** Characteristics of the Study Participants

Variable	All respondents (*N* = 191)	AS group (*N* = 151)	Surgical group (*N* = 40)	*p*-Value for comparison
Median age (IQR, years at time of questionnaire completion)	52 (43, 63)	55 (46, 68)	47 (37, 55)	0.002
Female sex, *n* (%)	147 (77)	118 (78)	29 (72)	0.590
Marital status at time of enrollment, *n* (%)				
Single	24 (13)	20 (13)	4 (10)	0.009
Married	144 (75)	108 (72)	36 (90)	
Divorced, separated, or widowed	23 (12)	23 (15)	0 (0)	
Had children at time of enrollment, *n* (%)	152 (80)	120 (79)	32 (80)	1.00
College/university of higher education level at time of enrollment, *n* (%)				
Did not finish high school	16 (8)	16 (11)	0 (0)	<0.001
High school degree completed	32 (17)	31 (21)	1 (3)	
College or university degree	105 (55)	79 (52)	26 (65)	
Postgraduate or professional degree	38 (20)	25 (17)	13 (32)	
Employment status at time of enrollment, *n* (%)				
Paid employment	123 (64)	94 (62)	29 (72)	0.070
Student	6 (3)	4 (3)	2 (5)	
Full-time homemaker or unpaid caregiver	12 (6)	8 (5)	4 (10)	
Unemployed	6 (3)	4 (3)	2 (5)	
Retired	42 (22)	39 (26)	3 (7)	
Not working due to disability	2 (1)	2 (1)	0 (0)	
Race, *n* (%)				
White	112 (59)	90 (60)	22 (55)	0.740
Black	5 (3)	4 (3)	1 (3)	
Asian	58 (30)	46 (30)	12 (30)	
Hispanic	1 (1)	1 (1)	0 (0)	
Middle Eastern	11 (6)	7 (5)	4 (10)	
Indigenous	1 (1)	1 (1)	0 (0)	
Mixed	3 (2)	2 (1)	1 (3)	
Median baseline size of primary tumor (largest dimension in mm, IQR])	11 (9, 14)	11 (8, 13)	13 (9, 15)	0.010
Presence of multiple thyroid nodules at baseline, *n* (%)	139 (73)	108 (72)	31 (78)	0.580
Taking any prescribed thyroid medical treatment at the time of enrollment,^a^ *n* (%)				
Levothyroxine	24 (13)	16 (11)	8 (20)	0.670
Methimazole	3 (2)	3 (2)	0 (0)	
Natural desiccated thyroid hormone	1 (1)	1 (1)	0 (0)	

^a^ Prescribed thyroid medical treatment includes thyroid hormone (any type of prescription preparation) or antithyroid drugs and each is, respectively, described in this comparison.

AS, active surveillance; IQR, interquartile range.

### Thyroid cancer treatment status and clinical outcomes at follow-up

The mean duration of follow-up was 14 months (SD 4) in the AS group and 16 months (SD 5) in the surgical group (*p* = 0.005). At the time of the questionnaire follow-up, 7.2% (11/151) of respondents who initially chose AS had crossed over to definitive thyroid cancer treatment (10 surgery and 1 radiofrequency ablation). The median time from enrollment to crossing over from AS to surgery/definitive treatment was 8.7 months (interquartile range 7.8, 12.7 months). Of the 11 respondents who crossed over from AS to definitive treatment, 2 patients had disease progression, specifically tumor enlargement. The other nine patients who crossed over did so as per their own choice (without disease progression). One patient who crossed over from AS due to personal preference, chose to undergo radiofrequency ablation for a 12 mm PTC outside of the study.

The age of the patients significantly differed according to the treatment status: respondents who crossed over from AS to surgery—mean age 45.2 years (SD 12.1), respondents who remained under AS—mean age 55.8 years (SD 14.8), and respondents who initially chose surgery—mean age 46.8 years (SD 12.5) (*p* < 0.001 for the difference among groups). There was no significant sex difference between the respondents who crossed over from AS to surgery (73%, 8/11 female), and those who remained under AS (79%, 110/140) or those who initially chose surgery (73%, 29/40) (*p* = 0.65).

The relevant clinical treatments and one-year outcomes of patients are compared between groups in Table 2. Of the patients who had surgery or definitive treatment in the study, only a minority underwent total thyroidectomy (18%, 9/11 in the AS crossover group and 15%, 6/40 in the group who chose initial surgery, Table 2). Of the patients who underwent thyroid surgery, 20% in each group (AS crossover group 2/10 and surgical group 8/40) had any nodal dissection, with these largely consisting of central neck dissections (whereas one patient in the surgical group had a central and lateral neck dissection). At this point in follow-up, there were no patients who had permanent hypoparathyroidism requiring calcitriol treatment. However, there was one patient in the immediate surgery group who had surgery for hematoma evacuation after thyroidectomy and another patient in the surgical group who had temporary recurrent laryngeal nerve paresis (which subsequently recovered).

**Table 2.** Summary of Clinical Treatments and Outcomes of Participants at Follow-Up

Variable	All respondents (*N* = 191)	AS group (*N* = 151)	Surgical group (*N* = 40)	*p*-Value for comparison
Median duration of follow-up completed (months) (IQR)	14 (12, 16)	13 (12, 16)	15 (13, 18)	0.005
Type of initial thyroid surgery or other definitive treatment (of 51 procedures)				
Hemithyroidectomy	40/51 (78%)	7/11 (64%)	33/40 (83%)	0.130
Total thyroidectomy	8/51 (16%)	2/11 (18%)	6/40 (15%)	
Other	3/51 (6%)	2/11 (18%)^a^	1/40 (3%)^b^	
Taking any prescribed thyroid medical treatment^c^ at the time of 1-year assessment, *n* (%)				
Thyroid hormone	46 (24)	19 (13)	27 (68)	<0.001
Methimazole	2 (1)	2 (1)	0 (0)	
None	143 (75)	130 (86)	13 (32)	
Mean TSH level at 1-year follow-up assessment (mIU/L, SD) (*N* = number of patients from whom data are available)	1.65 (1.07) (*N* = 185)	1.64 (1.02) (*N* = 148)	1.67 (1.27) (*N* = 37)	0.930
Radioactive iodine remnant ablation or adjuvant radioactive iodine treatment received	4 (2%)	1 (1%)^d^	3 (8%)^e^	0.030
T of TNM Stage for patients who had surgery and histologic confirmation of diagnosis of thyroid cancer^f^ (*N* = 10 AS, *N* = 39 surgery)				
T1a	19/49 (39%)	5/10 (50%)	14/39 (36%)	0.480
T1b	30/49 (61%)	5/10 (50%)	25/39 (64%)	
N of TNM Stage for patients who had surgery and histologic confirmation of diagnosis of thyroid cancer^f^ (*N* = 10 AS, *N* = 39 Surgery)				
N0	35/49 (71%)	6/10 (60%)	29/39 (74%)	0.390
Nx	6/49 (12%)	1/10 (10)	5/39 (13%)	
N1a	8/49 (16%)	3/10 (30%)	5/39 (13%)	

^a^ One patient underwent initial isthmectomy and another patient underwent radiofrequency ablation. The patient who underwent isthmectomy developed disease recurrence and was treated with completion thyroidectomy and radioactive iodine adjuvant treatment.

^b^ One patient underwent isthmectomy.

^c^ Prescribed thyroid medical treatment includes thyroid hormone (any type of prescription preparation) or antithyroid drugs. The type of thyroid hormone being taken was levothyroxine, with the exception of one patient in the active surveillance group who was taking natural desiccated thyroid hormone (which was continued as it was started before the patient entering the study).

^d^ Dose activity of 70 mCi of radioactive iodine in one patient who crossed over to surgery and experienced disease recurrence.

^e^ The dose activity of radioactive iodine was ∼100 mCi for two patients and 30 mCi for one patient.

^f^ Excludes one patient in the surgical group who had surgery and there was no evidence of thyroid cancer and one patient in the active surveillance arm who underwent radiofrequency ablation.

SD, standard deviation; TSH, thyrotropin.

Of the 40 respondents who underwent surgery, 98% (39/40) had histologic evidence of PTC. One patient in the surgical group preoperative cytology suspicious for PTC had no histopathologic evidence of thyroid malignancy on hemithyroidectomy. One patient in the AS group who crossed over by choice and was treated with isthmectomy had disease recurrence (growing malignant nodule in a remaining lobe). This recurrence was treated with surgery and radioactive iodine remnant ablation, with no further evidence of structural disease at the time of questionnaire follow-up. No patients in the surgical group experienced disease recurrence by the time of the one-year follow-up questionnaire.

### Patient-reported outcomes

At follow-up, the mean level of decision regret was 22.4 (SD 13.9) in patients who chose AS and 20.9 (SD 12.2) in patients who chose surgery (*p* = 0.73) (Table 3). After adjusting for age, sex, and duration of follow-up, there was no significant difference in the level of decision regret between patients who chose AS or surgery (*p* = 0.29) (Table 3). In a secondary analysis examining decision regret according to the one-year follow-up treatment status, the mean level of decision regret was as follows: 21.5 (SD 12.8) in 140 patients remaining under AS, 33.5 (SD 22.2) in 11 patients who had crossed over from choosing AS and had surgery, and 20.9 (SD 12.2) in 40 patients who chose surgery initially (*p* = 0.12 for the comparison between groups).

**Table 3.** Patient-Reported Outcomes at One-Year Follow-Up (Overall Scores)

Variable	AS group (*N* = 151)	Surgical group (*N* = 40)	Comparison crude data (*p*)	Comparison adjusted data*^a^* (*p*)
Decision Regret Scale total score (SD)^b^	22.4 (13.9)	20.9 (12.2)	0.73	0.29
Fear of Progression Short Form, mean total score (SD)^c^	23.1 (8.7)	24.4 (9.8)	0.52	0.94
HADS Anxiety subscale score, mean (SD)^d^	4.7 (3.9)	6.8 (4.1)	0.003	0.09
HADS Depression subscale score, mean (SD)^d^	3.1 (3.4)	4.4 (4.0)	0.06	0.37
MD Anderson Symptom Inventory for Thyroid Cancer—Symptom Severity Subscale, mean (SD)^e^	0.8 (1.0)	1.3 (1.4)	0.09	0.07
MD Anderson Symptom Inventory for Thyroid Cancer—Symptom Interference (with life) Subscale, mean score (SD)^e^	0.9 (1.6)	1.8 (2.2)	0.009	0.02
BIS, mean total score (SD)^f^	2.4 (3.7)	4.2 (5.6)	0.06	0.18

^a^ Linear regression analysis adjusted for age, sex, and duration of follow-up.

^b^ The Decision Regret scale is scored on a scale from 0 to 100, where 100 represents the maximal (worst) level of regret.

^c^ The Fear of Progression (of disease)—Short Form total score may range from 5 to 60 (where 5 is the least fear and 60 is the most fear).

^d^ The Hospital Anxiety and Depression Scale respective subscales for anxiety and depression are scored on a scale from 0 to 21, where a higher score indicates worse symptoms.

^e^ The MD Anderson Symptom Inventory for Thyroid Cancer includes a Symptom Severity Subscale (13 items) and a Symptom Interference Subscale (6 items). The questions in each of these subscales are scored on a scale of 0 to 10, where 10 is the worst. The score of each subscale is estimated by averaging the score for each of the items included in the subscale.

^f^ The Body Image Scale includes 10 questions and the scores for each of the questions are summed, such that the total score may range from 0 (best, no symptoms/distress) to 30 (worst symptoms/distress).

BIS, Body Image Scale; HADS, Hospital Anxiety and Depression Scale.

Compared with patients who remained under AS, the level of decision regret was significantly higher in patients who crossed over from AS to surgery (beta coefficient 10.1 [CI 1.3–18.9], *p* = 0.02), but not in those who initially chose surgery (beta coefficient −1.4 [CI −6.5 to 3.6], *p* = 0.58, adjusted linear regression analysis).

There was no significant difference between patients who chose AS and those who chose surgery regarding the crude scores on the following questionnaires: Fear of Disease Progression, HADS depression subscale (HADS-D), the MDASI-Thy Symptom Severity Subscale, or the BIS score at one-year follow-up (Table 3). However, the mean level of anxiety measured on the HADS-A subscale (*p* = 0.003) was significantly greater in patients who chose surgery compared with those who chose AS (Table 3). Furthermore, individuals in the surgical group reported greater symptom interference with life functioning as per the MDASI-Thy Interference subscale, compared with patients who chose AS (*p* = 0.009) (Table 3), which was largely attributed to self-reported impairments in general activity, mood, work, relationship with other people, and enjoyment of life (Supplementary Table S1). Furthermore, on the BIS questionnaire, the mean scores were worse in the surgical group compared with the AS group for patient perceptions of the treatment as leaving the “body less whole” (*p* = 0.01) and dissatisfaction with the surgical scar appearance (*p* < 0.001) (Supplementary Table S2).

After adjusting for age, sex, and of follow-up duration, the finding of the MDASI-Thy symptom interference scale score remained significantly worse in the surgical group compared with the AS group (*p* = 0.02) (Table 3). However, the adjusted analyses for the global scores on the other patient-reported outcome questionnaires (Fear of Progression Questionnaire, HADS-A, HADS-D, MDASI-Thy Symptom Severity Subscale and BIS) did not demonstrate significant differences between groups (Table 3). After adjusting for age, sex, and follow-up duration, the level of anxiety as measured by HADS-A was not significantly different between the groups (*p* = 0.09, Table 3). *Post hoc* analyses of patient-reported outcome data according to ultimate treatment status at year 1 are shown in Supplementary Table S3.

### Change in fear of disease progression over time

**FIG. 2.**
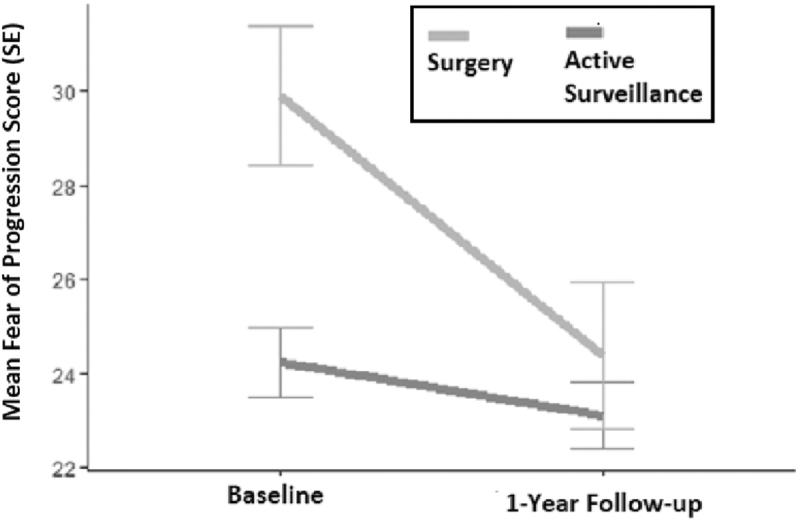
Change in fear of progression (of disease). SE, standard error.

At baseline, the mean Fear of Progression questionnaire score was significantly higher (worse) in the respondents in the surgical group 29.9 (SD 9.5) compared with the AS group (24.2, SD 9.1) (*p* < 0.001). However, at the one-year follow-up assessment, the Fear of Progression score did not significantly differ between groups: mean 23.1 (SD 8.7) in the AS group, and mean 24.4 (SD 9.8) in the surgical group in comparing crude data (*p* = 0.52) or in the analysis adjusted for age, sex, and follow-up duration (*p* = 0.94; Table 3). This finding was explained by a significantly greater reduction in fear of disease progression over the year of follow-up in the surgical group (mean difference −5.5, SD 9.7), compared with the AS group (−1.2, SD 7.6), since enrollment (*p* = 0.01) (Fig. 2).

## Discussion

In this prospective observational study evaluating patients with small, low-risk PTC, there was no significant difference in the mean level of decision regret between patients who chose AS or surgery. This finding is important to consider in the context of the participants' original fears relating to their disease and its treatment, as well as their disease management goals. We previously reported qualitative and quantitative data from our study showing that the participants who chose AS were more fearful of surgery (and its potential consequences), whereas those who chose surgery were more fearful of their malignancy and had a strong desire to remove cancer from their body.^16,27^ After one year, we observed that most patients in the AS group succeeded in their original goal to avoid surgery.

Furthermore, patients in the surgical group succeeded in having the cancer removed from their body, although with some perceived symptom burden attributed to treatment (e.g., appetite, drowsiness, sadness, feeling left “less whole,” and living with a surgical scar). The relatively low levels of decision regret in both the surgical and AS patient groups at follow-up may be a reflection of most patients avoiding the consequence that they feared the most (i.e., the disease versus the treatment), and those in the surgical arm were willing to accept some potential treatment-related symptom burden to rid themselves of cancer. Moreover, overall, symptom burden and anxiety scores were generally relatively low in our study.

In a secondary adjusted analysis, we observed that patients who crossed over from AS to surgery appeared to have more decision regret, but this finding is not unexpected as regret is generally associated with decision reversal^28^ and requires longer term follow-up. This research highlights the importance of understanding thyroid cancer patients' disease management goals, their fears, and providing well-informed disease management options, whenever possible.

Some of our findings confirm those observed internationally in patients with low-risk papillary microcarcinoma. A greater burden of physical symptoms/impairment in physical health (e.g., neck, scar or other symptoms) in patients with papillary microcarcinoma who have had surgery, compared with those who were under AS, has been reported by investigators from: Kuma Hospital in Japan,^29^ the MAeSTro study group from Korea,^30,31^ investigators from Tokyo, Japan,^32,33^ and investigators from Asan Medical Center in Korea.^34^ Investigators from China who studied patients with highly suspicious nodules 1 cm or smaller on ultrasound (with mandatory thyroid biopsy of patients in the surgical group but optional biopsy in the AS group) also reported a greater physical symptom burden in patients who had surgery compared with those under AS, with some scar-related symptoms persisting to 12 to 18 months after surgery.^35^

Published reports in the literature are variable regarding psychological health outcomes of patients with papillary microcarcinoma under AS compared with surgery. Similar to our observations, Jeon et al. from Korea reported no significant difference in the level of fear of disease progression in patients who were under AS (*N* = 43) compared with those who had a lobectomy (*N* = 148) about 2 to 3 years after diagnosis.^34^ Some recent cross-sectional studies from Japan have reported that anxiety levels were worse in patients who had surgery compared with those under AS.^29,33^ Kazusaka et al. reported that patients who had surgery had greater trait anxiety compared with those under AS.^33^ The MAeSTro study group from Korea reported that the psychological health in patients under AS was superior to that of patients who had surgery, when evaluated after about two years of prospective follow-up.^31^

Liu et al. reported that in Chinese patients with suspicious nodules 1 cm or smaller, there was no significant difference in psychological health parameters in patients who underwent AS or surgery, although not all of the patients under AS underwent thyroid biopsy.^35^ The level of anxiety in our study was relatively low among participants, which would make it difficult to find any statistically significant difference between groups, in context of a limited study size.

Some strengths of our study include the prospective design and a high participant response rate. Some limitations of our study include lack of randomization of participants to AS or surgery, baseline differences between study groups, and limited study population size. Our results may not necessarily be generalizable to other practice settings, as this study was conducted in a tertiary care center with specialized expertise in AS of small low-risk PTC. It is important to indicate that many of our participants were highly educated and it is possible that patient-reported outcomes could differ in individuals of other educational backgrounds. Lastly, the results of our secondary analyses should be considered hypothesis-generating and require confirmation in future prospective research.

Finally, in this study of patients with small, low-risk PTC, the mean level of decision regret pertaining to the initial disease management choice did not differ significantly between patients who chose AS or surgery. These results may be a reflection of participants being well-informed about their disease management options and their disease management goals being largely achieved. Further prospective research is needed to confirm and better understand the long-term, quality-of-life implications of the choice of AS or surgery for small, low-risk PTC.
