# Supplementary material for: Decision Regret Following the Choice of Surgery or Active Surveillance for Small, Low-Risk Papillary Thyroid Cancer: A Prospective Cohort Study
Source: Thyroid. 2024 May 24;34(5):626–34. doi: 10.1089/thy.2023.0634 (PMC11296158; doi:10.1089/thy.2023.0634)
Supplement: Supplementary Data [file thy.2023.0634_SupplementR1Dec212023clean.docx]

**Supplemental Methods:**

**Active Surveillance Option Description Pamphlet**

**
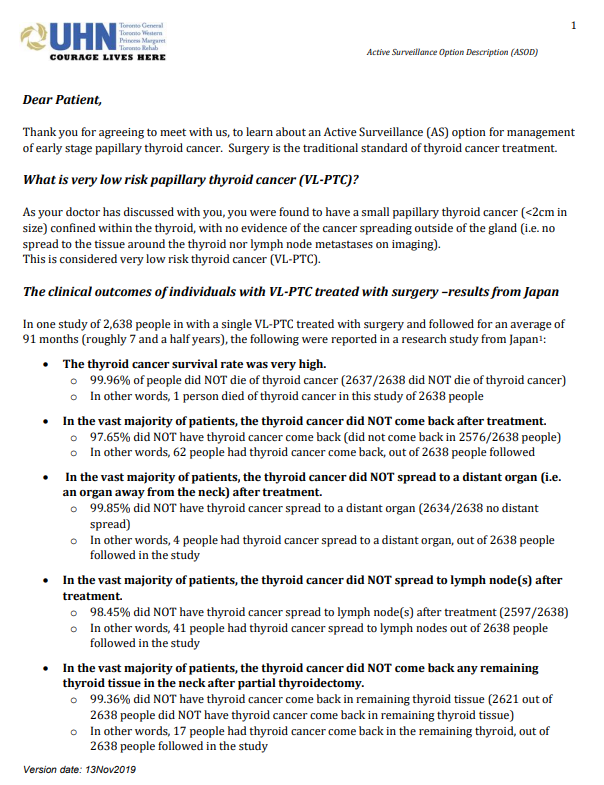
**

**
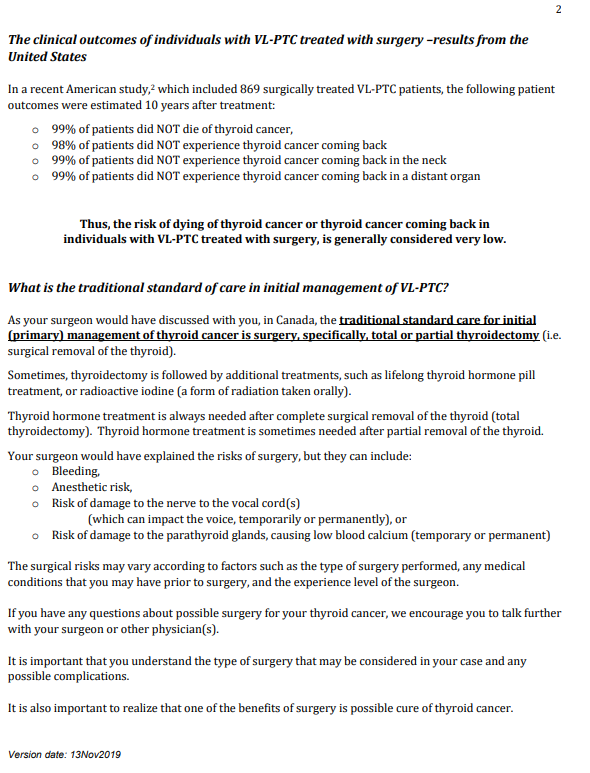
**

**
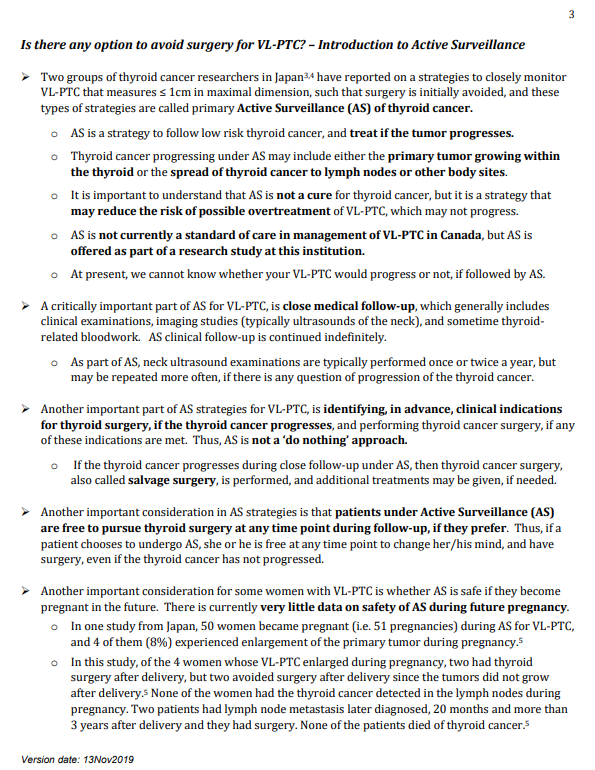
**

**
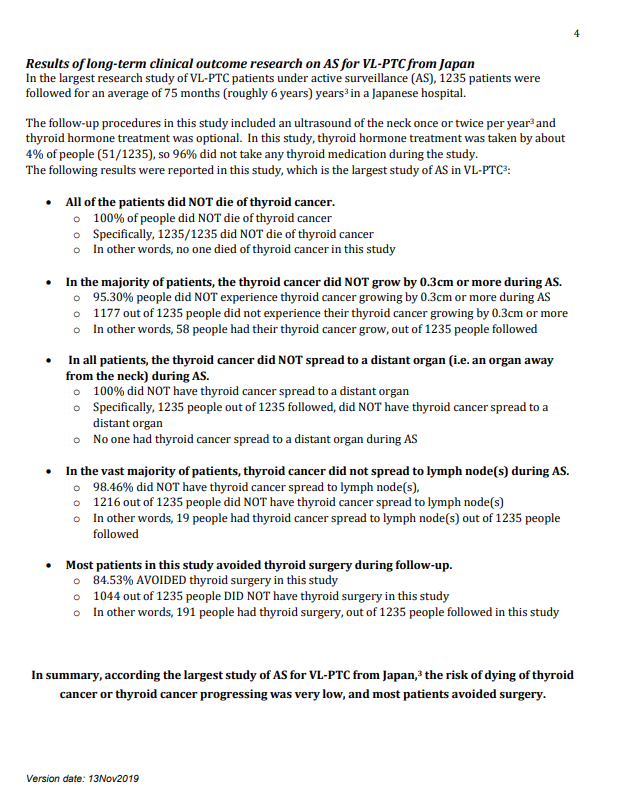
**

**
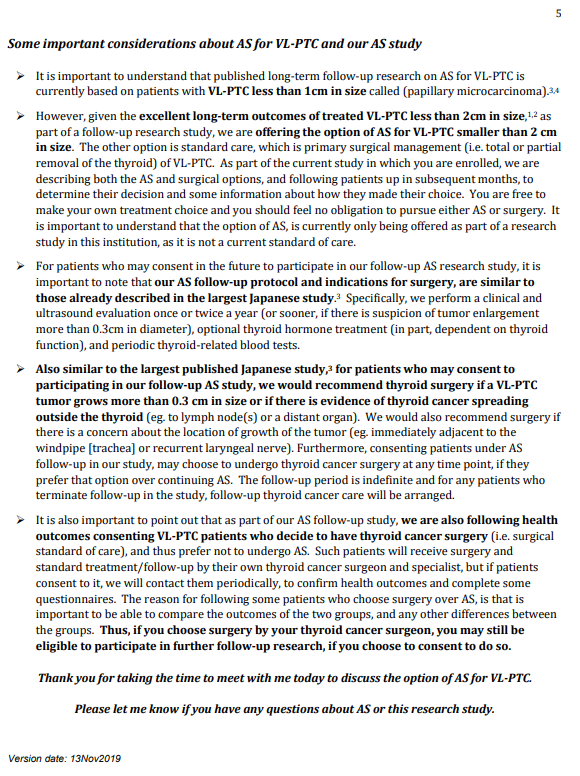
**

**
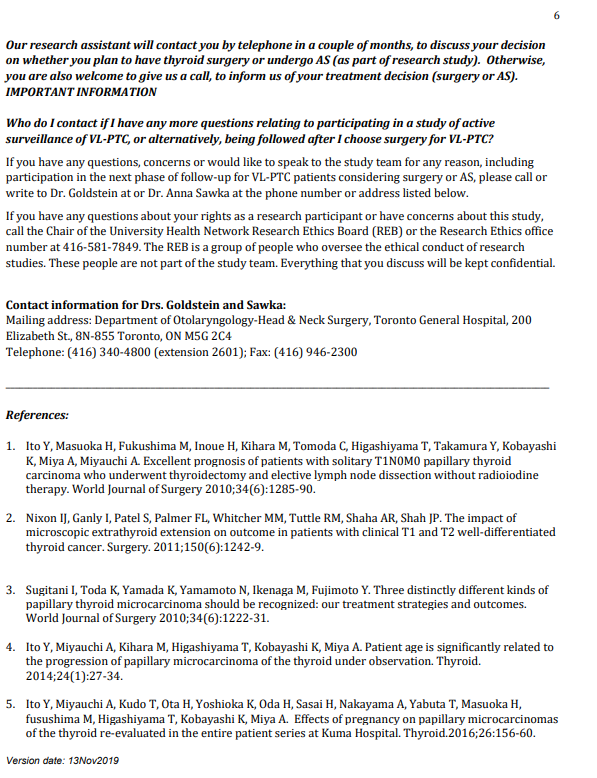
**

**Supplemental Table 1. Results of the MD Anderson Symptom Inventory Questionnaire**

1. **Symptom Severity Subscale**

Participants were instructed to rate the severity of their symptoms in the last 24 hours on a scale from from 0 (symptom has not been present) to 10 (the symptom was as bad as you can imagine it could be).

|  |  |  |  |
| --- | --- | --- | --- |
| **Question** | **Active Surveillance**  **(N = 151)**  **Mean (Standard Deviation, SD)** | **Surgery**  **(N = 40)**  **Mean (SD)** | **Comparison**  **(p-value)** |
| **CORE ITEMS** | | | |
| 1. Your **pain** at its WORST? | 0.27 (0.93) | 0.78(1.99) | 0.05 |
| 2. Your **fatigue (tiredness)** at its WORST? | 1.69 (2.38) | 2.65 (2.95) | 0.05 |
| 3. Your **nausea** at its WORST? | 0.23 (0.81) | 0.68 (1.88) | 0.16 |
| 4. Your **disturbed sleep** at its WORST? | 1.74 (2.57) | 1.83 (2.50) | 0.70 |
| 5. Your feeling of being **distressed (upset)** at its WORST? | 1.23 (2.13) | 1.70 (2.36) | 0.20 |
| 6. Your **shortness of breath** at its WORST? | 0.74 (1.50) | 1.00 (2.29) | 0.81 |
| 7. Your problem with **remembering things** at its WORST? | 1.47 (2.26) | 1.98 (2.37) | 0.10 |
| 8. Your problem with **lack of appetite** at its WORST? | 0.41 (1.10) | 0.93 (1.70) | **0.005** |
| 9. Your feeling **drowsy (sleepy)** at its WORST? | 1.35 (2.18) | 2.33 (2.79) | **0.001** |
| 10. Your having a **dry mouth** at its WORST? | 0.95 (1.73) | 1.48 (3.06) | 0.90 |
| 11. Your feeling **sad** at its WORST? | 1.06 (1.95) | 1.90 (2.62) | **0.04** |
| 12. Your **vomiting** at its WORST? | 0.07 (0.51 | 0.10 (0.30) | 0.05 |
| 13. Your **numbness or tingling** at its WORST? | 0.49 (1.36) | 0.70 (1.59) | 0.21 |
| **THYROID-SPECIFIC SYMPTOMS** | | | |
| 14. Your **hoarseness** at its WORST? | 0.38 (0.88) | 0.80 (1.89) | 0.29 |
| 15. Your **problem with feeling hot** at its WORST? | 1.29 (2.39) | 0.90 (1.72) | 0.96 |
| 16. Your **problem with racing heartbeat** at its WORST? | 0.76 (1.63) | 1.15 (2.08) | 0.59 |
| 17. Your **problem with feeling cold** at its WORST? | 0.99 (1.96) | 1.43 (2.25) | 0.12 |
| 18. Your **difficulty swallowing** at its WORST? | 0.46 (1.09) | 0.93 (2.25) | 0.63 |
| 19. Your **diarrhea or loose stools** at its WORST? | 0.53 (1.54) | 0.58 (1.26) | 0.27 |

1. **Symptom Interference Subscale**

Participants were instructed to rate how much their symptoms interfered with their life functioning in the last 24 hours on a scale from from 0 (symptom has not been present) to 10 (the symptom was as bad as you can imagine it could be).

|  |  |  |  |
| --- | --- | --- | --- |
| **Question** | **Active Surveillance**  **(N = 151)**  **Mean (Standard Deviation, SD)** | **Surgery**  **(N = 40)**  **Mean (SD)** | **Comparison**  **(p-value)** |
| **20. General activity?** | 0.83 (1.77) | 1.93 (2.65) | **0.002** |
| **21. Mood?** | 1.09 (1.97) | 2.20 (2.69) | **0.003** |
| **22. Work (including work around the house)?** | 0.99 (2.00) | 1.90 (2.42) | **0.002** |
| **23. Relations with other people?** | 0.85 (1.83) | 1.50 (2.11) | **0.02** |
| **24. Walking?** | 0.75 (1.76) | 1.35 (2.41) | 0.09 |
| **25. Enjoyment of life?** | 1.02 (1.97) | 1.80 (2.46) | **0.02** |

**Supplemental Table 2. Results of the Body Image Scale Questionnaire**

In this questionnaire, participants asked how they felt about their appearance, and about any changes that may have resulted from their thyroid cancer or its treatment. Each item was scored on a likert scale, with scores ranging from 0 (not at all) to 3 (very much).

| **Statement** | **Active Surveillance**  **(N = 151)**  **Mean (Standard Deviation, SD)** | **Surgery**  **(N = 40)**  **Mean (SD)** | **Comparison**  **(p-value)** |
| --- | --- | --- | --- |
| 1. Have you been feeling self-conscious about your appearance? | 0.62 (0.83) | 0.78(0.92) | 0.32 |
| 1. Have you felt less physically attractive as a result of your disease or treatment? | 0.23 (0.49) | 0.50 (0.85) | 0.05 |
| 1. Have you been dissatisfied with your appearance when dressed? | 0.33 (0.60) | 0.53 (0.75) | 0.09 |
| 1. Have you been feeling less feminine/masculine as a result of your disease or treatment? | 0.14 (0.43) | 0.23 (0.53) | 0.28 |
| 1. Did you find it difficult to look at yourself naked? | 0.29 (0.62) | 0.35 (0.74) | 0.78 |
| 1. Have you been feeling less sexually attractive as a result of your disease or treatment? | 0.18 (0.48) | 0.38 (0.71) | 0.05 |
| 1. Did you avoid people because of the way you felt about your appearance? | 0.17 (0.48) | 0.20 (0.56) | 0.88 |
| 1. Have you been feeling the treatment has left your body less whole? | 0.11 (0.37) | 0.30 (0.61) | 0.01 |
| 1. Have you felt dissatisfied with your body? | 0.34 (0.64) | 0.45 (0.68) | 0.24 |
| 1. Have you been dissatisfied with the appearance of your scar? (from thyroid cancer surgery)* | 1.05 (0.21) | 1.450(0.82) | <0.001 |

*For question 10, not applicable responses in patients who did not have thyroid surgery were scored as “0”

**Supplemental Table 3. Post-hoc analyses of patient reported outcomes according to 1-year treatment status**

| **Variable at One-year follow-up assessment** | **Continuing Active Surveillance**  **(N = 140)** | **Crossed Over from Active Surveillance to Definitive Treatment (N = 11)** | **Chose Surgery**  **(N = 40)** | **Comparison**  **crude data**  **(p-value)** |
| --- | --- | --- | --- | --- |
| **Decision Regret Scale total score (standard deviation, SD)*** | 21.5 (12.8) | 33.5 (22.2) | 20.9 (12.2) | 0.12 |
| **Fear of Progression Short Form– Mean total score (SD)†** | 23.2 (8.9) | 22.0 (5.8) | 24.4 (9.8) | 0.810 |
| **Hospital Anxiety and Depression Scale (HADS) Anxiety subscale score – mean (SD)‡** | 4.6 (3.8) | 6.6 (4.5) | 6.8 (4.1) | 0.003 |
| **HADS Depression subscale score – Mean (SD)‡** | 2.9 (3.2) | 5.9 (3.8) | 4.4 (4.0) | 0.008 |
| **MD Anderson Symptom Inventory for Thyroid Cancer – Symptom Severity Subscale – Mean (SD)Ø** | 0.9 (1.0) | 0.8 (0.7) | 1.3 (1.4) | 0.170 |
| **MD Anderson Symptom Inventory for Thyroid Cancer – Symptom Interference (with life) Subscale – Mean score (SD)Ø** | 0.9 (1.6) | 1.3 (1.6) | 1.8 (2.2) | 0.008 |
| **Body Image Scale – Mean total score (SD)§** | 2.2 (3.6) | 5.0 (3.7) | 4.2 (5.6) | 0.002 |

πLinear regression analysis adjusted for age, sex, and duration of follow-up

*The Decision Regret scale is scored on a scale from zero to 100, where 100 represents the maximal (worst) level of regret

†The Fear of Progression (of disease) – Short form total score may range from 5 to 60 (where 5 is the least fear and 60 is the most fear).

‡The Hospital Anxiety and Depression Scale respective subscales for Anxiety and Depression are scored on a scale from 0 to 21, where a higher score indicates worse symptoms.

ØThe MD Anderson Symptom Inventory for Thyroid Cancer includes a Symptom Severity Subscale (13 items) and a Symptom Interference Subscale (6 items). The questions in each of these subscales are scored on a scale of 0 to 10, where 10 is the worst. The score of each subscale is estimated by averaging he score for each of the items included in the subscale.

§The Body Image Scale includes 10 questions and the scores for each of the questions are summed, such that the total score may range from 0 (best, no symptoms/distress) to 30 (worst symptoms/distress).
